# Effectiveness of therapeutic exercises for improving the quality of life of patients with chronic venous insufficiency: a systematic review

**DOI:** 10.1590/1677-5449.200248

**Published:** 2021-06-16

**Authors:** Josicléia Leôncio da Silva, Ana Gonçalves Lima, Natália Ramos Diniz, Jéssica Costa Leite

**Affiliations:** 1 Centro Universitário Unifacisa – UNIFACISA, Campina Grande, PB, Brasil.; 2 Universidade Estadual da Paraíba – UEPB, Campina Grande, PB, Brasil.

**Keywords:** chronic venous insufficiency, therapeutic exercises, quality of life, pain, physical functional performance

## Abstract

The main signs and symptoms of chronic venous insufficiency are pain, edema, varicose veins, and tissue changes; conditions that compromise functionality and quality of life. Management of the disease aims to mitigate these losses and involves a wide range of interventions, one of which is therapeutic exercise. This article presents the existing evidence on the effectiveness of therapeutic exercises for quality of life, pain, and functionality in chronic venous insufficiency. Searches were run on the databases CENTRAL, CINAHL, LILACS, MEDLINE, PEDro, SciELO, Science Direct, Scopus, and Web of Science. Four of the 2,961 results met the eligibility criteria. Only one of these studies showed benefits of exercise for improving quality of life and reducing pain. The others had low methodological quality. The existing evidence is therefore insufficient to indicate or contraindicate therapeutic exercises for improvement of quality of life, pain, and functionality in patients with chronic venous insufficiency.

## INTRODUCTION

Chronic venous disease (CVD) is defined as any dysfunction that affects venous system homeostasis and results in appearance of signs and/or symptoms ranging from simple telangiectasia to severe ulcerations.^1^ In turn, the term chronic venous insufficiency (CVI) is used to refer to venous disease involving the lower limbs.^2^ Ulceration is a component of CVI that characterizes the most advanced stage of the disease.^3^

Lower limb CVI is a disease that is primarily associated with venous hypertension combined with other factors, such as valve incompetence. The consequence of these changes is to provoke blood stasis.^4^ This disease affects approximately 30% of the global population and the main signs and symptoms related to it are pain, edema, throbbing, feelings of heaviness in the limbs, itching, varicose veins, and tissue changes.^5^

All of these clinical characteristics have direct impact on quality of life (QoL), especially the intensity of pain, severity of edema, and presence of inflammation.^6^ It should be noted that functionality and QoL are intimately related phenomena, since the QoL of people with CVI reduces as impairment of physical activity levels and functional capacity increases, which in turn worsens the prognosis of the disease.^7^

Treatment for CVI can be invasive or conservative.^8^ Conservative approaches include physiotherapy, which treats and prevents the complications of CVI by means of compression therapy, lymphatic drainage, hydrotherapy, and therapeutic exercises.^9^ The exercise protocols employed generally combine flexibility, strength, and resistance training, with the objective of strengthening the peripheral muscle pumps and improving venous return.^10^

Against this background, the present study was conducted to attempt to answer the following question: Are interventions with therapeutic exercises in patients with CVI effective at improving these individuals’ QoL? The study objective was therefore to present the best scientific evidence level demonstrating the effectiveness of therapeutic exercises for treatment of people with CVI, highlighting the available clinical evidence of its effects on QoL (primary outcome), and on pain and functionality.

## METHODS

### Research setting

Searches were run on the databases MEDLINE (Medical Analysis and Retrieval System Online); LILACS (Literature Latino‐Americana and do Caribe em Ciências da Health); SciELO (Scientific Electronic Library Online); Scopus (Elsevier); CINAHL (The Cumulative Index to Nursing and Allied Health Literature); Web of Science (now part of Clarivate Analytics); CENTRAL (The Cochrane Central Register of Controlled Trials); Science Direct, and PEDro (Physiotherapy Evidence Database). Google Scholar was also used to identify articles in the “gray literature”, using the same search strategy used for the other databases. Manual searches were also conducted in the references of articles selected for full-text reading and in previously published reviews, seeking any other potentially eligible studies. All searches were run in English with no publication language or date restrictions.

The search strings were constructed using MeSH (Medical Subject Headings) and DeCS (Descritores em Ciências da Saúde) keywords combined with the Boolean operators “AND” and “OR”. The combinations employed were adapted to meet the requirements of each database and the keywords used were: “Venous Insufficiency”, “Exercise Therapy”, “Therapeutic Exercise”, “Quality of Life”, Pain, “Physical Performance”, “Physical Functional Performance”, and “Clinical Trial”.

### Inclusion and exclusion criteria

Randomized clinical trials (RCTs) and crossover studies conducted with adult human beings (> 18 years) with CVI were selected for the study. These studies used therapeutic exercises (aerobics, lower limb flexibility training and/or lower limb strength training) as primary or adjuvant intervention, compared with other interventions (control group) or no treatment. The studies also analyzed the effects of exercises on QoL, pain, and/or functionality. Studies were excluded if the abstract or full text was not available, if they were study protocols or ongoing studies, or if they had not conducted a baseline assessment or post-intervention assessment.

### Data collection instruments and procedures

Initially, the PICO strategy was employed (Table 1). PICO is an acronym for population (P), which comprises individuals with CVI; intervention (I), the therapeutic exercises; comparison (C), with other treatments or control groups; and outcomes (O), for which the primary outcome was QoL, and secondary outcomes were pain and functionality. Four search strategies were used and search strings labeled #1, #2, #3, and #4 were developed to enact them. These strings had to be modified for two of the databases (CINAHL and PEDro), as shown in Table 2.

**Table 1:** PICO Strategy.

**PICO Strategy**	**Corresponding keyword**
P (venous insufficiency)	“Venous Insufficiency”
I (therapeutic exercises)	“Therapeutic Exercise”, “Exercise Therapy”, “Exercise Therapy”
C (control group)	
O (QoL, pain, and functionality)	“Quality of Life”, “Pain”, “Physical Functional Performance”, “Physical Performance”

PICO = Patients, Intervention, Comparison and Outcomes; QoL = quality of life.

**Table 2:** Detailed description of search strings employed.

**Database**	**Strategy**	**Search strings used in searches**
CINAHL^*^	#1	“Venous Insufficiency” AND “Therapeutic Exercise” AND “Quality of Life”
	#2	“Venous Insufficiency” AND “Therapeutic Exercise” AND Pain
	#3	“Venous Insufficiency” AND “Therapeutic Exercise” AND “Physical Performance”
	#4	“Venous Insufficiency” AND “Therapeutic Exercise”
CENTRAL, Google Scholar, LILACS, MEDLINE, SciELO, Science Direct, Scopus, Web of Science	#1	“Venous Insufficiency” AND “Exercise Therapy” AND “Quality of Life”
	#2	“Venous Insufficiency” AND “Exercise Therapy” AND Pain
	#3	“Venous Insufficiency” AND “Exercise Therapy” AND “Physical Functional Performance”
	#4	“Venous Insufficiency” AND “Exercise Therapy” OR Exercise
PEDro*	-	Venous Insufficiency AND Clinical Trial

* Databases for which search strings had to be adapted.

The article selection process took place during September and October of 2019. Searches were performed by two researchers independently (JLS and AGLN). Initially, studies were selected by title, then by abstract and, finally, by reading of the full text and application of the eligibility criteria. The two researchers’ results were compared afterwards. In cases of divergent decisions, a third investigator (JCL) with more experience was contacted to facilitate the discussions.

### Data extraction

Data were extracted from eligible articles. General information about each study was noted, including authors’ names, years of publication, research setting, objectives, sample characteristics, eligibility criteria, methodology, interventions, prescription of exercises, assessment instruments, outcomes, methods used for analysis of the results, conclusions, etc.

### Study quality assessment

Risk of bias assessment was conducted according to the Cochrane Handbook for Systematic Reviews of Interventions. This assessment covers seven domains, each of which is classified as high risk of bias, low risk of bias, or uncertain risk of bias.^11^ Any assessment disagreements were resolved in discussions with the third investigator.

In the current review, the following aspects were assessed: random allocation sequence generation (selection bias), allocation concealment (selection bias), implementation of the control group (performance bias), blinding of participants (performance bias), blinding of outcome assessment (detection bias), incomplete outcome data (attrition bias), and selective outcome reporting (data reporting bias). Additionally, the scientific evidence level and the recommendation grade by study type were also assessed, as recommended by the Oxford Center for Evidence-based Medicine.

## RESULTS AND DISCUSSION

### Selection of studies

**Figure 1:**
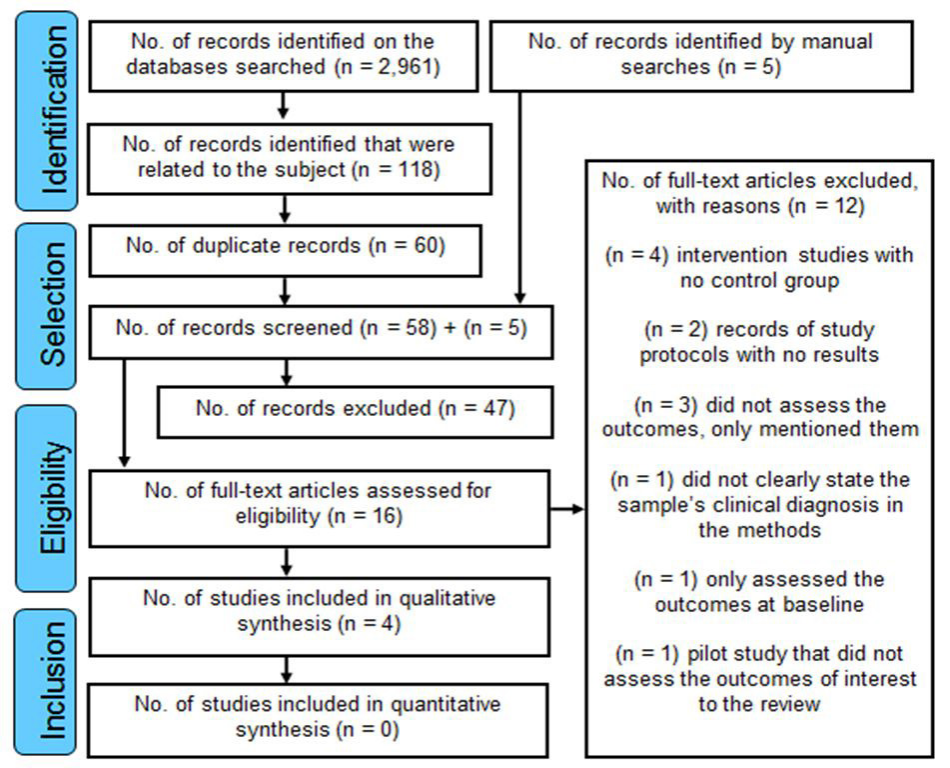
Preferred Reporting Items for Systematic Reviews and Meta-Analyses (PRISMA) flow diagram illustrating selection of studies. Adapted from Moher et al.^12^

The searches returned a total of 2,961 results, distributed as follows: MEDLINE, 268; LILACS, 441; SciELO, 208; Scopus, 378; CINAHL, 14; Web of Science, 7; CENTRAL, 46; Science Direct, 962; PEDro, 35; and Google Scholar, 602. However, only four publications met the eligibility criteria and were chosen for a qualitative synthesis, since none of the articles fulfilled the necessary criteria for quantitative comparison by meta-analysis. The search and selection process is illustrated in a Preferred Reporting Items for Systematic Reviews and Meta-Analyses (PRISMA) flow diagram,^12^ shown in Figure 1.

The samples studied in four articles selected comprised a total of 189 volunteers, 98 of whom were allocated at random to experimental groups and 91 of whom were allocated to control groups. These patients’ Clinical, Etiological, Anatomic, and Pathological (CEAP) disease classifications varied from C1 to C6, with symptoms ranging from varicose veins to ulcerations. With regard to sex distribution, 116 volunteers were female (61.38%) and 73 were male (38.62%), while mean age of study groups ranged from 62.15±11.23 to 72.3±10.13 years.

The outcomes of interest to this review were investigated as primary or secondary outcomes in the studies. The most prevalent types of exercises employed were flexibility, strength, and resistance. All of the articles were published in English, but the studies were conducted in four different countries: the United States, Poland, Spain, and Australia.

It is noteworthy that one of the studies^13^ was conducted with postmenopausal women only. The authors explained this choice with the claim that CVI is most prevalent in this population. Another study^14^ only enrolled men, but did not explain the reason for this choice and the information was only presented in the footnote to a table. The main findings of the studies are shown in Table 3.

**Table 3:** Characteristics of the studies selected.

**Author, year, and country**	**Characteristics of the sample**	**Intervention and duration**	**Outcomes assessed and results**
Padberg et al.,^14^ 2004, United States	Total (n = 30), male, CEAP (4, 5 and 6).	EG: compression therapy, individualized exercises and educational pamphlet.	Function, strength and resistance of the muscle pump; venous hemodynamics; AMA; functionality; and QoL.
	EG: (n = 17), mean age of 71 years.	CG: compression therapy only (30-40 mmHg).	There were no differences in QoL or functional mobility, but improvements were observed in hemodynamics, function, and muscle pump strength.
	CG: (n = 13), mean age of 70 years.	- 6 months.	
Szewczyk et al.,^15^ 2010, Poland	Total (n = 32), men (n = 11), women (n = 21), CEAP (6).	EG: compression therapy, special dressings, supervision and instructions on exercises to be performed at home.	AMA; ulcer area, pain, and clinical scores for patients with ulcerations.
	EG: (n = 16), men (n = 4), women (n = 12), age = 72.2±7.66 years.	CG: the same treatment, but unsupervised and with the exception of exercise on an exercise bicycle.	Pain associated with physical activity was observed in both groups, but intensity was not correlated with AMA. Ankle joint mobility improved in both groups, but was more significant in the experimental group. Ulcer area and lipodermatosclerosis exhibited a significant effect on AMA.
	CG: (n=16), Men (n=7), Women (n=9), Age= 72.3 ± 10.13 years.	- 9 weeks.	
Ramos-González et al.,^13^ 2012, Spain	Total (n = 65), female, CEAP (1 and 2).	EG: kinesiotherapy, myofascial release (50 min., 2x per week) and instructions on exercises to be performed at home.	Arterial blood pressure, QoL, venous circulation; and pain.
	EG: (n = 33), age = 65.75±9.07 years.	CG: kinesiotherapy only.	The combination of these treatments improved symptoms of pain, QoL, arterial blood pressure, and venous return blood flow.
	CG: (n = 32), age = 62.15±11.23 years.	- 10 weeks.	
O’Brien et al.,^16^ 2017, Australia	Total (n = 62), men (n = 32), women (n = 30), CEAP (6).	EG: resisted exercises, compression therapy, wound care, educational pamphlet, telephone coaching, and instructions on exercises to be performed at home.	Ulcer healing and area, physical activity, functional capacity, QoL, and AMA.
	EG: (n = 32), age = 71.3±15.8 years.	CG: usual care and educational pamphlet on care for the feet.	All participants exhibited improvements in physical activity levels. Healing was significant in the exercises group. No differences were found in QoL or functional capacity, but there were good AMA results in the experimental group.
	CG: (n = 30), age = 71.7±13.4 years.	-12 weeks.	

CEAP = Clinical, etiological, anatomic, and pathological classification; n = total number of participants; EG = experimental group; CG = control group; min. = minutes; mmHg = millimeters of mercury; AMA = ankle movement amplitude; QoL = quality of life.

There were considerable differences in the methodology employed in each RCT in terms of execution, sample size, exercise modality, sessions, series, repetitions, duration, intensity, frequency, follow-up of volunteers, and assessment and analysis of outcomes. These variations impact on analysis of the results observed and prevent their replication.

With regard to the number of participants, 75% of the studies^13^^,^^15^^,^^16^ had a sample size exceeding thirty volunteers. It is of note that only two of the studies reported a sample size calculation: one study^14^ should have recruited a sample of 60 participants, but only achieved 50% of this number, while the other^16^ should have recruited 110 volunteers, but was conducted with 62 (56.36%).

With regard to the variables of interest to this review, each study assessed one or a maximum of two outcomes using a variety of different instruments and at specific times (Table 4). This variation in assessment tools was one of the reasons that quantitative comparison or meta-analysis of the results were not possible.

**Table 4:** Summary of study outcomes and assessment instruments and intervals.

**Studies**	**Outcomes**	**Assessment instruments**	**Assessments**
Padberg et al.^14^	-QoL;	-AVVQ;	Baseline and after 6 months.
	-Functionality.	-CIVIC;	
		-SF-36;	
		-MFI.	
Szewczyk et al.^15^	-Pain.	-10-point numerical scale^*^;	Baseline, every week, and after 9 weeks.
		-Clinical signs and symptoms scale for patients with ulcerations*.	
Ramos-González et al.^13^	-QoL;	-VAS;	Baseline and after 10 weeks.
	-Pain.	-SF-36.	
O’Brien et al.^16^	-QoL;	-SF-8;	Baseline and after 12 weeks.
	-Functionality.	- Tinetti Gait and Balance Test.	

QoL = quality of life; AVVQ = Aberdeen Varicose Veins Questionnaire; CIVIC = Chronic Venous Insufficiency Questionnaire; SF-36 = Short Form Health Survey; MFI = mean functional independence; VAS: visual analog scale; SF-8: Short Form-8.

* The authors did not provide a specific name for this instrument.

Although the exercise protocols differed, the therapeutic objectives were similar: to improve muscle pump function in the legs and boost venous return. There were also similarities in the preference for exercises focused on the ankle joint and the triceps surae muscles, which are important anatomic structures involved in CVI.

Two RCTs^14^^,^^15^ used flexibility, strength, and resistance exercises that were supplemented with aerobic exercises, while another study^13^ used the same three modalities (flexibility, strength, and resistance), but supplemented with breathing exercises. The protocol adopted in the fourth study^16^ comprised flexibility, strength, and resistance exercises only.

With regard to specific information on the methodology adopted during performance of exercises, none of the studies described the protocol completely and there were several information gaps, making the interpretation needed for an adequate comparative analysis impossible. Only one of the studies^16^ provided details about the protocol adopted, but still failed to provide information on the number of series, repetitions, duration, and progression of exercises (Table 5).

**Table 5:** Description of the exercise protocols used in the studies selected.

**Study**	**Exercises protocol**
Padberg et al.^14^	Stretching and strength exercises for the trunk and lower limbs (primarily the triceps surae muscles) performed actively against gravity and weight resistance; walking on a treadmill.
	- One hour of exercises. Series and repetitions were increased progressively.
Szewczyk et al.^15^	Circular ankle movements; heel raises in standing position; dorsal flexion; plantar flexion. Exercise bicycle (twice a week) at moderate intensity for 20 min. and 3 km walking daily.
	- 3 times per day, in series of 15 repetitions each.
Ramos-González et al.^13^	Flexion and extension of the metacarpophalangeal and interphalangeal joints, with participant sitting; flexion and extension of the ankle, with participant in standing position; isometric contraction of the quadriceps muscles with the knees extended and participant sitting; diaphragmatic respiration.
	- Twice a day, every day.
O’Brien et al.^16^	Heel raises, in sitting position, both lower limbs; heel raises, in standing position, both lower limbs; heel raises, one limb at a time, in standing position; stretches (for 20 seconds) of the triceps surae and hamstring muscles of both lower limbs, before and after exercises.
	- 3 times per day, every day. Series and repetitions were increased progressively.

min.: minutes; km: kilometers.

### Study quality and publication bias

**Figure 2:**
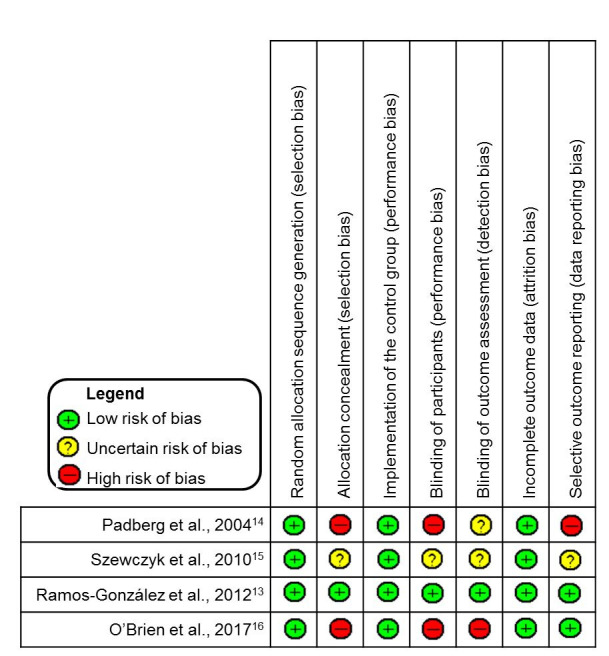
Analysis of risk of bias of studies, according to the Cochrane Handbook for Systematic Reviews of Interventions.^17^

According to the Oxford Center for Evidence-based Medicine criteria, only one of the studies^13^ presented sufficient clinical evidence to achieve a Level 1 assessment, and was classified with recommendation grade A and evidence level 1B. The other three had Level 2 evidence ratings, classified at recommendation grade B and evidence level 2B (low quality RCTs).^14^^-^16 The quality of the studies was also assessed in terms of their risk of bias, and each item analyzed was given one of the following classifications: low risk of bias, uncertain risk of bias, or high risk of bias^17^ (Figure 2).

The studies were classified as low risk of bias for random allocation sequence generation because they used computer generated random numbers,^13^^,^^16^ coin tossing,^15^ and a standardized random number table (list).^14^ For allocation concealment, two studies were considered to have high risk of bias because they used an open randomization process based on a list of numbers^14^ or because they did not conceal allocation.^16^ The third study^15^ was judged to have uncertain risk of bias because of missing information and just one study^13^ was classified as low risk of bias, because in addition to using an allocation center, lots were folded and sealed and placed in opaque envelopes.

Only one study^13^ could be considered low risk of bias for blinding of participants, because its participants did not know which group they belonged to. Two studies^14^^,^^16^ were classified as having high risk of bias, because volunteers were not blinded, and one RCT^15^ did not present clear information on this matter.

With regard to blinding of outcome assessment, only one of the studies^13^ blinded the evaluator of outcome variables. Two studies^14^^,^^15^ did not provide information on who was responsible for outcome assessments. The fourth study^16^ was considered to have a high risk of bias because the evaluator was not blinded, which could have interfered with the results of the outcomes.

For the item incomplete outcome data, two studies^13^^,^^15^ did not report losses from the sample during follow-up, while the other two studies^14^^,^^16^ did. However, the authors justified these losses, claiming that they had not interfered in the data relating to the outcomes assessed. These studies were considered as having low risk of bias.

**Figure 3:**
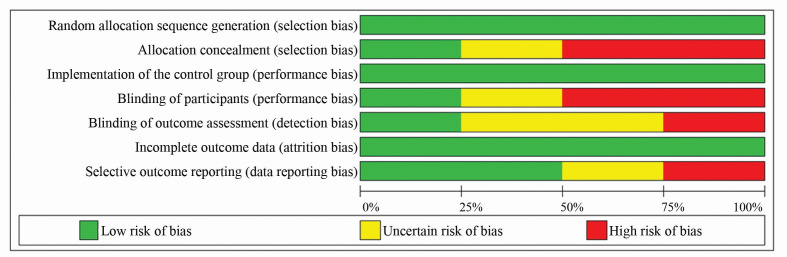
Graph illustrating risk of bias according to the Cochrane Handbook for Systematic Reviews of Interventions.

With regard to selective outcome data, only one study^14^ was classed as having a high risk of bias, because the authors did not show data on some of the variables proposed, claiming that no significant differences had been observed. The second study^15^ did not provide sufficient information to assess this issue, and the other two studies^13^^,^^16^ detailed the results for all of the variables proposed. Figure 3 shows a summary of the results of the RCT quality assessment based on analysis of their risks of bias.

### Effects on quality of life

The study by Padberg et al.^14^ detected no significant differences in the outcome QoL, even though the study employed assessment instruments specifically developed for people with CVI. In contrast, Ramos-González et al.^13^ did find evidence of significant results (p = 0.047) for QoL, but the intervention adopted combined exercises with myofascial release therapy.

The authors’ explanation for the positive results was that the technique employed acted directly on the muscle fascia, an important component in propulsion of venous return. The hypothesis was that myofascial release therapy would eliminate fascial restrictions, stretching the muscle and reestablishing its physiological length, enabling it to function well, and improving venous return. As a consequence, edema and pain would be reduced, which would be reflected in QoL.^13^ The study conducted by O’Brien et al.^16^ did not detect any significant effect (p = 0.91) on volunteers’ QoL, but the authors claimed that the study had low power to detect an effect because of the small sample size and short intervention period.

### Effects on functionality

The RCT conducted by Padberg et al.^14^ did not detect any significant differences and the authors decided not to show the data on this variable. The study by O’Brien et al.^16^ observed a similar finding, in that the data analysis did not reveal any significant effect (p = 0.21). However, these authors highlight an interesting observation – overall physical activity levels improved in all of the study participants (p = 0.06).

With regard to this finding, the researchers reported that before randomization was conducted, all of the volunteers were given instructions on the exercises that would be performed. This could have had the effect that control group participants had incorporated the exercises into their own care habits on their own initiative.^16^

### Effects on pain

The study by Szewczyk et al.^15^ found a correlation between pain intensity level and ankle movement amplitude (AMA), but did not find any relevant associations. However, presence of pain associated with physical activity was an observation that was present in all study participants. In contrast, Ramos-González et al.^13^ did find statistically significant results for reduction in pain, both when this variable was compared among the members of the experimental group (p = 0.035) and when compared against the scores for the control group (p = 0.038).

### Limitations

The findings of this review show that just one of the studies reported that therapeutic exercises had benefits for participants’ QoL. However, this information is not definitive, taking into consideration the small sample investigated, although the study did have a low risk of bias. It is also important to emphasize that the systematic review itself is subject to certain limitations that should be considered.

One important limitation affecting the review was the fact that there are few RCTs. With regard to the studies that were selected, there were certain failures related to the exercise protocols adopted, since crucial information on the intensity, frequency, number of series, repetitions, duration, and progression of exercises either was not provided or was incomplete.

Additionally, the heterogeneous nature of the samples (number of participants, absence or inadequacy of sample size calculations, and sex) rules out extrapolation of the results to the population with CVI and also replication of the protocol by other researchers. Moreover, divergences in terms of analysis and presentation of outcomes meant that the results obtained could not be grouped to conduct quantitative analysis using meta-analysis.

## CONCLUSIONS

Only one of the four RCTs identified reported positive and significant results, attributed to the effects of the therapeutic exercises on the QoL of the participants assessed. However, the quality of the evidence reported in the studies that exist on therapeutic exercises in CVI is weak or uncertain. This makes it impossible to confirm its efficacy on QoL, functionality, and pain.

Therefore, there is insufficient evidence to indicate or to contraindicate therapeutic exercises for improvement of QoL, pain, and functionality in patients with CVI. This finding underscores the need for additional research, adopting greater methodological rigor to limit the possibility of biases to a minimum.
