# Supplementary material for: Effectiveness of therapeutic exercises for improving the quality of life of patients with chronic venous insufficiency: a systematic review
Source: J Vasc Bras. 2021 Jun 16;20:e20200248. doi: 10.1590/1677-5449.200248 (PMC8218828; doi:10.1590/1677-5449.200248)
Supplement: Translation

# Translation: Eficácia dos exercícios terapêuticos na qualidade de vida de pacientes com insuficiência venosa crônica: uma revisão sistemática

**Authors:** Josicléia Leôncio da Silva, Ana Gonçalves Lima, Natália Ramos Diniz, Jéssica Costa Leite

**Affiliations:** 1 Centro Universitário Unifacisa – UNIFACISA, Campina Grande, PB, Brasil.; 2 Universidade Estadual da Paraíba – UEPB, Campina Grande, PB, Brasil.

**Keywords:** insuficiência venosa crônica, exercícios terapêuticos, qualidade de vida, dor, desempenho físico funcional

## Abstract

Os principais sinais e sintomas da insuficiência venosa crônica são dor, edema, varizes e alterações teciduais, condições que comprometem a funcionalidade e qualidade de vida. Visando amenizar esses prejuízos, o manejo da doença envolve uma ampla modalidade de intervenções; entre elas, o exercício terapêutico. Esta pesquisa apresenta as evidências existentes sobre a efetividade dos exercícios terapêuticos na qualidade de vida, dor e funcionalidade da insuficiência venosa crônica. Efetuou-se uma busca nas bases de dados CENTRAL, CINAHL, LILACS, MEDLINE, PEDro, SciELO, Science Direct, Scopus e Web of Science. Dos 2.961 resultados, quatro atenderam aos critérios de elegibilidade. Desses, apenas um estudo mostrou benefícios dos exercícios para melhora da qualidade de vida e redução da dor. Os demais apresentaram baixa qualidade metodológica. Portanto, as evidências existentes são insuficientes para indicar ou contraindicar os exercícios terapêuticos para melhoria da qualidade de vida, dor e funcionalidade em pacientes com insuficiência venosa crônica.

## INTRODUÇÃO

Define-se como doença venosa crônica (DVC) toda e qualquer disfunção que afete a homeostasia do sistema venoso resultando no aparecimento de sinais e/ou sintomas, que incluem desde simples telangiectasias até graves ulcerações^1^. Já o termo insuficiência venosa crônica (IVC) é utilizado para caracterizar a doença venosa que acomete os membros inferiores (MMII)^2^, sendo as ulcerações um componente que caracteriza o estágio mais avançado da doença^3^.

A IVC de MMII é uma doença que está envolvida principalmente com a hipertensão venosa associada a outros fatores, como a insuficiência valvular. A consequência dessas alterações consiste na formação de estase sanguínea^4^. A doença afeta aproximadamente 30% da população mundial adulta, e os principais sinais e sintomas relacionados a ela são dor, edema, latejamento, sensação de peso no membro, prurido, varizes e alterações teciduais^5^.

Todas essas características clínicas interferem diretamente na qualidade de vida (QV), especialmente a intensidade dolorosa, gravidade do edema e presença de inflamação^6^. Vale ressaltar que a funcionalidade e a QV são condições que se relacionam intimamente, pois a QV das pessoas com IVC diminui conforme há aumento do comprometimento dos níveis de atividade física e da capacidade funcional, o que piora o prognóstico da doença^7^.

No que se refere ao tratamento, ele pode ser invasivo ou conservador^8^. Entre as abordagens conservadoras, inclui-se a fisioterapia, que trata e previne as complicações da IVC por meio de terapia compressiva, drenagem linfática, hidroterapia e exercícios terapêuticos^9^. Geralmente, os protocolos de exercícios adotados integram treino de flexibilidade, força e resistência, objetivando potencializar a bomba muscular periférica e favorecer o retorno venoso^10^.

Diante do exposto, o presente estudo pretende responder à seguinte questão: a intervenção com exercícios terapêuticos em pacientes com IVC é eficaz para promover a melhoria da QV desses indivíduos? Assim, o objetivo desta pesquisa foi demostrar, através do melhor nível de evidência científica, a efetividade dos exercícios terapêuticos no tratamento de pessoas com IVC, enfatizando o que há de evidência clínica a respeito de seus efeitos sobre a QV (desfecho principal), assim como na dor e na funcionalidade.

## MÉTODOS

### Cenário da pesquisa

Realizou-se uma busca nas bases de dados MEDLINE (*Medical Analysis and Retrieval System Online*); LILACS (Literatura Latino‐Americana e do Caribe em Ciências da Saúde); SciELO (*Scientific Electronic Library Online*); Scopus (Elsevier); CINAHL (*The Cumulative Index to Nursing and Allied Health Literature*); Web of Science (atual *Clarivate Analytics*); CENTRAL (*The Cochrane Central Register of Controlled Trials*); Science Direct e PEDro (*Physiotherapy Evidence Database*). Além disso, a página virtual do Google Acadêmico foi utilizada visando selecionar artigos por meio da “literatura cinzenta”, adotando-se os mesmos cruzamentos das outras bases. Também foi realizada uma busca manual nas referências dos artigos selecionados para leitura completa e das revisões já existentes à procura de quaisquer outros estudos potencialmente elegíveis. Todas as buscas foram realizadas em inglês, sem restrição de idioma ou ano de publicação.

Para selecionar os termos e montar as estratégias de busca, foram utilizados o MeSH (*Medical Subject Headings*) e o DeCS (Descritores em Ciências da Saúde), assim como os operadores booleanos “*AND*” e “*OR*”. Os cruzamentos obedeceram às exigências de cada base, em que os descritores controlados utilizados foram: “*Venous Insufficiency*”, “*Exercise Therapy*”, “*Therapeutic Exercise*”, “*Quality of Life*”, *Pain*, “*Physical Performance*”, “*Physical Functional Performance*” e “*Clinical Trial*”.

### Critérios de inclusão e exclusão

Fizeram parte desta pesquisa ensaios clínicos randomizados (ECRs) e estudos cruzados (*crossover*) com seres humanos adultos (> 18 anos) portadores de IVC. Tais pesquisas utilizaram como intervenção principal ou complementar os exercícios terapêuticos (aeróbicos, flexibilidade de MMII e/ou de fortalecimento de MMII) comparados a outras intervenções (grupo-controle) ou nenhum tratamento. Além disso, os estudos incluídos analisaram os efeitos dos exercícios sobre a QV, dor e/ou funcionalidade. Foram excluídas pesquisas que não disponibilizavam o resumo ou texto na íntegra, protocolos de pesquisas e estudos em andamento ou aqueles que não realizaram as avaliações na linha de base e após a aplicação da intervenção.

### Instrumentos e procedimentos para coleta de dados

Inicialmente, utilizou-se a estratégia denominada pelo acrônimo PICO (Tabela 1), em que o P (*population*) foi representado pelos indivíduos com IVC; I (*intervention*), os exercícios terapêuticos; C (*comparation*) incluiu outros tratamentos ou grupo-controle; e O (*outcomes*) teve como desfecho principal QV, e os secundários foram dor e funcionalidade. As estratégias de cruzamentos utilizadas foram denominadas simbolicamente de #1, #2, #3 e #4. Algumas bases exigiram adequações (CINAHL e PEDro) para que as buscas pudessem ser efetuadas, conforme exemplificado na Tabela 2.

**Tabela 1:** Estratégia PICO.

**Estratégia PICO**	**Descritores correspondentes**
P (insuficiência venosa)	“*Venous Insufficiency*”
I (exercícios terapêuticos)	“*Therapeutic Exercise*”, “*Exercise Therapy*”, “*Exercise Therapy*”
C (grupo-controle)	
O (QV, dor e funcionalidade)	“*Quality of Life*”, *“Pain”*, “*Physical Functional Performance*”, “*Physical Performance*”

PICO = Pacientes, Intervenção, Comparação e *Outcomes*; QV = qualidade de vida.

**Tabela 2:** Descrição detalhada dos cruzamentos utilizados.

**Banco de dados**	**Estratégia**	**Cruzamento adotado nas buscas**
CINAHL^*^	#1	“*Venous Insufficiency*” *AND* “*Therapeutic Exercise*” *AND* “*Quality of Life*”
	#2	“*Venous Insufficiency*” *AND* “*Therapeutic Exercise*” *AND Pain*
	#3	“*Venous Insufficiency*” *AND* “*Therapeutic Exercise*” *AND* “*Physical Performance*”
	#4	“*Venous Insufficiency” AND “Therapeutic Exercise*”
CENTRAL, Google Acadêmico, LILACS, MEDLINE, SciELO, Science Direct, Scopus, Web of Science	#1	“*Venous Insufficiency” AND “Exercise Therapy” AND “Quality of Life”*
	#2	“*Venous Insufficiency” AND “Exercise Therapy” AND Pain*
	#3	“*Venous Insufficiency” AND “Exercise Therapy” AND “Physical Functional Performance”*
	#4	“*Venous Insufficiency” AND “Exercise Therapy” OR Exercise*
PEDro*	-	*Venous Insufficiency AND Clinical Trial*

* Bases em que foram realizadas adaptações dos cruzamentos.

A seleção dos artigos ocorreu em setembro e outubro de 2019. As buscas foram executadas por duas pesquisadoras (JLS e AGLN) independentes. Inicialmente, os estudos foram selecionados pelo título, depois, pelo resumo e, por fim, realizou-se a leitura completa, aplicando os critérios de elegibilidade. Posteriormente, os resultados foram comparados. Em caso de divergências, uma terceira revisora (JCL), com mais experiência, foi contatada para auxiliar nas discussões.

### Extração dos dados

Os artigos elegíveis tiveram seus dados extraídos. Foram coletadas informações gerais sobre o estudo, como nome do autor, ano de publicação, local da pesquisa, objetivos, características da amostra, critérios de elegibilidade, metodologia, intervenções, prescrição de exercícios, instrumentos avaliativos, desfechos, método de análise dos resultados, conclusões etc.

### Avaliação da qualidade dos estudos

Realizou-se a avaliação do risco de viés conforme o Manual Cochrane para Revisões Sistemáticas de Intervenções. Essa avaliação compreende sete domínios, em que cada um recebe uma classificação, podendo ser alto risco de viés, baixo risco de viés ou risco de viés incerto^11^. As discordâncias existentes quanto à avaliação foram resolvidas através de discussões realizadas com a terceira revisora.

Nesta revisão, estes aspectos foram avaliados: geração de sequência aleatória (viés de seleção), sigilo de alocação (viés de seleção), instalação de grupo-controle (viés de execução), cegamento dos participantes (viés de execução), mascaramento da avaliação dos resultados (viés de detecção), dados incompletos no resultado (viés de atrito) e informação seletiva de desfecho (viés de reportagem dos dados). Além disso, o nível de evidência científica e o grau de recomendação por tipo de estudo também foram avaliados, conforme o *Oxford Centre for Evidence-based Medicine*.

## RESULTADOS E DISCUSSÃO

### Seleção dos estudos

Foram identificados 2.961 resultados, divididos da seguinte forma: MEDLINE, 268; LILACS, 441; SciELO, 208; Scopus, 378; CINAHL, 14; Web of Science, 7; CENTRAL, 46; Science Direct, 962; PEDro, 35; e Google Acadêmico, 602. No entanto, apenas quatro publicações atenderam aos critérios de elegibilidade e foram eleitas para a síntese qualitativa, pois nenhum artigo atendeu aos critérios necessários para comparação quantitativa por metanálise. O processo de busca e seleção está exemplificado no fluxograma [*Preferred Reporting Items for Systematic Reviews and Meta-Analyses* (PRISMA)]^12^ da Figura 1.

**Figura 1:** Fluxograma da seleção dos estudos de acordo com o PRISMA (*Preferred Reporting Items for Systematic Reviews and Meta-Analyses*). Adaptado de Moher et al.^12^.

Dos quatro artigos selecionados, 189 voluntários fizeram parte das amostras. Desses, 98 foram randomizados aleatoriamente para o grupo experimental e 91, para o controle. A classificação Clínica, Etiológica, Anatômica e Patológica (CEAP) da doença variou entre C1 e C6, abordando desde varizes a ulcerações varicosas. Com relação ao sexo, 116 voluntários eram do sexo feminino (61,38%) e 73, masculino (38,62%); a média de idade variou de 62,15±11,23 a 72,3±10,13 anos.

Entre os desfechos de interesse desta revisão, as pesquisas os avaliaram de forma principal ou secundária. Quanto aos exercícios adotados, houve predominância dos de flexibilidade, força e resistência. No que diz respeito ao idioma, os artigos foram publicados na língua inglesa, porém as pesquisas ocorreram em países distintos: Estados Unidos, Polônia, Espanha e Austrália.

Vale ressaltar que um dos estudos^13^ foi realizado apenas com mulheres na pós-menopausa. Os autores justificaram essa escolha alegando que a IVC é mais prevalente nessa população. Outra pesquisa^14^ incluiu apenas homens, mas sem especificar o motivo da escolha, sendo essa informação relatada apenas no rodapé de uma tabela. Os principais achados dos estudos estão organizados na Tabela 3.

**Tabela 3:** Caracterização dos estudos selecionados.

**Autor, ano e país**	**Características da amostra**	**Intervenção e duração**	**Desfechos avaliados e resultados**
Padberg et al.^14^, 2004, Estados Unidos	Total (n = 30), sexo masculino, CEAP (4, 5 e 6).	GE: terapia compressiva, exercícios individualizados e cartilha educativa.	Função, força e resistência da bomba muscular; hemodinâmica venosa; ADM do tornozelo; funcionalidade e QV.
	GE: (n = 17), idade média de 71 anos.	GC: apenas terapia compressiva (30-40 mmHg).	Não houve diferenças na QV e mobilidade funcional, porém observou-se melhora na hemodinâmica, função e força da bomba muscular.
	GC: (n = 13), idade média de 70 anos.	- 6 meses.	
Szewczyk et al.^15^, 2010, Polônia	Total (n = 32), homens (n = 11), mulheres (n = 21), CEAP (6).	GE: terapia compressiva, curativo especial, acompanhamento e orientações sobre os exercícios que deveriam ser executados em casa.	ADM de tornozelo; área da úlcera, dor e escores clínicos de pacientes com ulcerações.
	GE: (n = 16), homens (n = 4), mulheres (n = 12), idade = 72,2±7,66 anos.	GC: a mesma terapia, porém sem supervisão e com exceção do exercício em bicicleta ergométrica.	A dor associada à atividade física foi evidenciada em ambos os grupos, porém não houve correlação da intensidade com a ADM de tornozelo. Houve melhora da mobilidade dessa articulação em ambos os grupos, porém foi mais significativa no grupo experimental. A área da úlcera e a lipodermatoesclerose apresentaram efeito significativo sobre a ADM do tornozelo.
	
	GC: (n=16), Homens (n=7), Mulheres (n=9), Idade= 72,3 ± 10,13 anos.	- 9 semanas.	
Ramos-González et al.^13^, 2012, Espanha	Total (n = 65), sexo feminino, CEAP (1 e 2).	GE: cinesioterapia, liberação miofascial (50 min., 2x por semana) e orientações sobre exercícios que deveriam ser executados em casa.	Pressão arterial, QV, circulação venosa e dor.
	GE: (n = 33), idade = 65,75±9,07 anos.	GC: apenas cinesioterapia.	A combinação dessas terapias melhorou os sintomas de dor, a QV, a pressão arterial e o fluxo sanguíneo do retorno venoso.
	GC: (n = 32), idade = 62,15±11,23 anos.	- 10 semanas.	
O’Brien et al.^16^, 2017, Austrália	Total (n = 62), homens (n = 32), mulheres (n = 30), CEAP (6).	GE: exercícios resistidos, terapia compressiva, cuidados com o ferimento, cartilha educativa, acompanhamento telefônico e orientações sobre os exercícios que deveriam ser executados em casa.	Cicatrização e área da úlcera, atividade física, capacidade funcional, QV e ADM do tornozelo.
	GE: (n = 32), idade = 71,3±15,8 anos.	GC: cuidados habituais e cartilha informativa sobre cuidados com os pés.	Todos os participantes apresentaram melhora nos níveis de atividade física. A cicatrização foi significativa no grupo de exercícios. Não foram evidenciadas diferenças na QV e capacidade funcional, porém houve bons resultados na ADM do tornozelo no grupo experimental.
	GC: (n = 30), idade = 71,7±13,4 anos.	-12 semanas.	

CEAP = classificação Clínica, Etiológica, Anatômica e Patológica; n = número total de participantes; GE = grupo experimental; GC = grupo-controle; min. = minutos; mmHg = milímetro de mercúrio; ADM = amplitude de movimento; QV = qualidade de vida.

Acerca da metodologia de cada ECR, existem diferenças consideráveis no que se refere a execução, tamanho amostral, modalidade de exercício, sessões, séries, repetições, duração, intensidade, frequência, seguimento dos voluntários, avaliação e análise dos desfechos. Essas variações interferem na análise dos resultados encontrados, inviabilizando sua reprodução.

Em relação ao número de participantes, 75% dos estudos^13^^,^^15^^,^^16^ apresentaram um tamanho amostral superior a trinta voluntários. Vale salientar que apenas duas pesquisas realizaram o cálculo amostral: um estudo^14^ deveria compor uma amostra de 60 participantes, porém só alcançou 50%, enquanto outro^16^ deveria ter 110 voluntários, mas a pesquisa foi realizada com 62 (56,36%).

No que diz respeito às variáveis que são de interesse desta revisão, cada pesquisa avaliou um ou no máximo dois desfechos com instrumentos diversos e em momentos específicos (Tabela 4). Essa variação das ferramentas de avaliação foi um dos motivos que inviabilizou a comparação quantitativa dos resultados encontrados, assim como a realização da metanálise.

**Tabela 4:** Síntese dos estudos conforme os desfechos, instrumentos e intervalo das avaliações.

**Estudos**	**Desfechos**	**Instrumentos de avaliação**	**Avaliações**
Padberg et al.^14^	-QV;	-AVVQ;	Linha de base e após 6 meses.
	-Funcionalidade.	-CIVIC;	
		-SF-36;	
		-MIF.	
Szewczyk et al.^15^	-Dor.	-Escala numérica de 10 graus^*^;	Linha de base, a cada semana, e após 9 semanas.
		-Escala clínica de sinais e sintomas de pacientes com ulcerações*.	
Ramos-González et al.^13^	-QV;	-VAS;	Linha de base e após 10 semanas.
	-Dor.	-SF-36.	
O’Brien et al.^16^	-QV;	-SF-8;	Linha de base e após 12 semanas.
	-Funcionalidade.	-Teste de *Tinetti Gait and Balance.*	

QV = qualidade de vida; AVVQ = *Aberdeen Varicose Veins Questionnaire*; CIVIC = *Chronic Venous Insufficiency Questionnaire;* SF-36 = *Short Form Health Survey*; MIF = medida de independência funcional; VAS: *visual analogue scal*e; SF-8: *Short Form*-8.

* O nome específico do instrumento não foi descrito pelos autores.

No que se refere aos protocolos de exercícios, apesar de serem distintos, os objetivos terapêuticos eram similares: melhorar a função da bomba muscular da perna e favorecer o retorno venoso. Também houve semelhança na preferência por exercícios direcionados à articulação do tornozelo e aos músculos tríceps sural, pois são importantes estruturas anatômicas envolvidas na IVC.

Dois ECRs^14^^,^^15^ utilizaram exercícios de flexibilidade, força e resistência que foram incrementados pelos exercícios aeróbicos, enquanto outro estudo^13^ empregou as mesmas modalidades (flexibilidade, força e resistência), porém acrescentou os exercícios respiratórios. Já em outra pesquisa^16^, o protocolo adotado incluiu apenas os exercícios de flexibilidade, força e resistência.

Quanto às informações específicas sobre a metodologia adotada durante a execução dos exercícios, as pesquisas não descreveram o protocolo por completo e muitas lacunas ficaram evidentes, dificultando a interpretação para uma adequada análise comparativa. Apenas um dos estudos^16^ forneceu detalhes sobre o protocolo adotado, porém ainda faltaram referências quanto ao número de séries, repetições, tempo e progressão dos exercícios (Tabela 5).

**Tabela 5:** Descrição dos protocolos de exercícios dos estudos selecionados.

**Estudo**	**Protocolo de exercícios**
Padberg et al.^14^	Alongamento e fortalecimento do tronco e dos MMII (principalmente dos músculos tríceps sural) realizados ativamente contra a gravidade e resistência de pesos; caminhada em esteira ergométrica.
	- Uma hora de exercícios. Houve progressão das séries e repetições.
Szewczyk et al.^15^	Movimentos circulares do tornozelo; elevação do calcanhar em ortostatismo; flexão dorsal; flexão plantar. Mais bicicleta ergométrica (2 vezes por semana) em intensidade moderada por 20 min. e caminhada diária de 3 km.
	- 3 vezes por dia, em séries de 15 repetições cada.
Ramos-González et al.^13^	Flexão e extensão das articulações metacarpofalângicas e interfalângicas, participante em sedestação; flexão e extensão do tornozelo, participante em ortostatismo; contração isométrica dos músculos quadríceps com os joelhos estendidos, participante em sedestação; respiração diafragmática.
	- 2 vezes por dia, todos os dias.
O’Brien et al.^16^	Elevação do calcanhar, em sedestação, ambos os MMII; elevação do calcanhar, em ortostatismo, ambos os MMII; elevação do calcanhar, um membro por vez, em ortostatismo; alongamentos (sustentados por 20 segundos) dos músculos tríceps sural e isquiotibiais, em ambos os MMII, antes e após os exercícios.
	- 3 vezes por dia, todos os dias. Houve progressão das séries e repetições.

MMII: membros inferiores; min.: minutos; km: quilômetro.

### Qualidade dos estudos e viés de publicação

De acordo com os critérios do *Oxford Center for Evidence-based Medicine*, apenas um estudo^13^ apresentou evidências clínicas suficientes para atingir uma distinção de Nível 1, sendo classificado com grau de recomendação A e nível de evidência 1B. As demais pesquisas apresentaram força de evidência de Nível 2, enquadrando-se em um grau de recomendação B, com nível de evidência 2B (ECRs de baixa qualidade)^14^^-^16. Além disso, os estudos tiveram sua qualidade avaliada conforme o risco de viés apresentado, e cada item analisado recebeu a seguinte classificação: baixo risco de viés, risco obscuro de viés ou alto risco de viés^17^ (Figura 2).

**Figura 2:** Análise do risco de viés dos estudos, conforme o Manual Cochrane para Revisões Sistemáticas de Intervenções^17^.

Quanto à geração de sequência aleatória, os estudos foram classificados com baixo risco de viés, pois utilizaram para alocação a geração de números randômicos por computador^13^^,^^16^, o arremesso de moedas^15^ e uma tabela (lista) numérica randômica padronizada^14^. Com relação ao sigilo de alocação, dois estudos foram considerados com alto risco de viés por utilizarem um processo aberto de randomização mediante uma lista numérica^14^ ou por não adotarem a alocação oculta^16^. Outra pesquisa^15^ foi avaliada com risco obscuro de viés, por ausência de informações. Apenas um estudo^13^ foi considerado com baixo risco de viés, pois, além de ter uma central de alocação, também utilizou fichas dobradas e seladas em envelopes opacos.

No que diz respeito ao cegamento dos participantes, apenas um estudo^13^ pode ser considerado com baixo risco de viés, pois os participantes não tiveram conhecimento sobre qual grupo pertenciam. Outras duas pesquisas^14^^,^^16^ foram avaliadas com alto risco de viés por não adotarem o cegamento dos voluntários, enquanto um dos ECRs^15^ não apresentou informações claras.

Sobre o mascaramento da avaliação dos resultados, em apenas um dos estudos^13^ o avaliador das variáveis estava cego. Outras duas pesquisas^14^^,^^15^ não relataram informações sobre quem foi o responsável pela avaliação dos desfechos. Em contrapartida, um estudo^16^ foi considerado com alto risco de viés porque o avaliador não estava cego, o que pode ter interferido nos resultados dos desfechos.

No que se refere aos dados incompletos no resultado, duas pesquisas^13^^,^^15^ não relataram perdas na amostra durante o seguimento, à medida que outros dois estudos^14^^,^^16^ relataram. No entanto, os autores justificaram os desfalques alegando que não houve interferência nos dados relacionados aos desfechos avaliados. Esses estudos foram considerados com baixo risco de viés.

No quesito relato de desfecho seletivo, apenas um estudo^14^ foi considerado com alto risco de viés porque os autores não apresentaram os dados de algumas variáveis propostas, alegando que não foram observadas diferenças significativas. Outro estudo^15^ não forneceu informações suficientes para avaliação, enquanto duas pesquisas^13^^,^^16^ retrataram nos resultados todas as variáveis propostas. Um resumo dos resultados da avaliação da qualidade dos ECRs desta revisão mediante a análise dos riscos de viés está retratado na Figura 3.

**Figura 3:** Gráfico do risco de viés de acordo com o Manual Cochrane para Revisões Sistemáticas de Intervenções.

### Efeitos na qualidade de vida

No estudo de Padberg et al.^14^, não houve diferenças significativas para o desfecho QV, apesar da pesquisa ter utilizado instrumentos de avaliação específicos para indivíduos com IVC. Entretanto, Ramos-González et al.^13^ evidenciaram a ocorrência de resultados significativos (p = 0,047) na QV, porém a intervenção adotada associou aos exercícios a terapia de liberação miofascial.

A justificativa dos autores para os resultados positivos seria a de que a técnica utilizada atua diretamente na fáscia muscular, um importante componente para propulsão do retorno venoso. A liberação miofascial eliminaria as restrições fasciais, alongando o músculo, reestabelecendo seu comprimento fisiológico, permitindo seu bom funcionamento e melhorando o retorno venoso. Consequentemente, ocorreria a redução do edema e da dor, refletindo na QV^13^. Já a pesquisa de O’Brien et al.^16^ não demostrou nenhum efeito significativo (p = 0,91) sobre a QV dos voluntários, porém os autores afirmaram que a potência do estudo para avaliar algum efeito foi baixa em virtude do pequeno tamanho da amostra e do curto período de intervenção.

### Efeitos sobre a funcionalidade

O ECR de Padberg et al.^14^ não encontrou diferenças significativas, e os autores decidiram não apresentar dados sobre essa variável. No estudo de O’Brien et al.^16^, foi encontrado um achado semelhante, no qual a análise dos dados não revelou nenhum efeito significativo (p = 0,21). Entretanto, os autores chamam atenção para um fato curioso, que foi a melhora dos níveis de atividade física geral de todos os participantes do estudo (p = 0,06).

Sobre esse ocorrido, os pesquisadores relataram que, antes da randomização, todos os voluntários receberam instruções sobre os exercícios que seriam realizados. Portanto, isso pode ter feito com que os participantes do grupo-controle tenham incluído, por conta própria, os exercícios na rotina dos cuidados habituais^16^.

### Efeitos sobre a dor

A pesquisa de Szewczyk et al.^15^ correlacionou o nível de intensidade dolorosa com a amplitude de movimento (ADM) de tornozelo, mas não encontrou nenhuma associação relevante. No entanto, a presença de dor associada à atividade física foi um dado presente em todos os participantes do estudo. Em contrapartida, Ramos-González et al.^13^ apresentaram resultados estatisticamente significativos para a redução da dor, tanto quando a variável foi comparada entre os integrantes do próprio grupo experimental (p = 0,035) quanto com os escores do grupo-controle (p = 0,038).

### Limitações

Os achados desta pesquisa demostraram que apenas um dos estudos mostrou os benefícios dos exercícios terapêuticos na QV dos participantes. No entanto, essa informação não é definitiva, levando-se em consideração a pequena amostra avaliada, apesar de o estudo ter apresentado baixo risco de viés. Também é importantes ressaltar que esta revisão sistemática apresenta algumas limitações que devem ser consideradas.

Uma importante limitação para esta revisão foi a existência de poucos ECRs. No que diz respeito aos estudos selecionados, ocorreram falhas nos protocolos de exercícios adotados, porque informações cruciais sobre intensidade, frequência, número de séries, repetições, tempo e progressão do exercício deixaram de ser fornecidas ou estavam incompletas.

Além disso, a heterogeneidade das amostras (quantidade de participantes, ausência ou inadequações do cálculo amostral e sexo) inviabiliza a ampliação dos resultados para a população com IVC, assim como a reprodução do protocolo por outros pesquisadores. Ainda, as divergências em relação a análise e exposição dos desfechos impossibilitaram o agrupamento dos resultados obtidos para que pudesse ser realizada uma análise quantitativa por metanálise.

## CONCLUSÕES

Apenas um dos quatro ECRs encontrados apresentou resultados positivos e significativos, atribuídos aos efeitos dos exercícios terapêuticos sobre a QV dos participantes avaliados; porém, a qualidade das evidências dos estudos existentes sobre os exercícios terapêuticos na IVC é fraca ou incerta. Isso impossibilitou a confirmação de sua eficácia sobre a QV, funcionalidade e dor.

Portanto, as evidências existentes são insuficientes para indicar ou contraindicar os exercícios terapêuticos para melhoria da QV, dor e funcionalidade em pacientes com IVC. Esse achado reforça a necessidade de que novas pesquisas sejam realizadas, adotando-se um maior rigor metodológico para reduzir ao máximo as possibilidades de vieses.
